# In Search for Equilibrium: Immunosuppression Versus Opportunistic Infection

**DOI:** 10.14740/jocmr2439w

**Published:** 2015-12-28

**Authors:** Tariq Yousuf, Jason Kramer, Adam Kopiec, Brody Jones, Joy Iskandar, Khansa Ahmad, Hesam Keshmiri, Muhyaldeen Dia

**Affiliations:** a Department of Internal Medicine, Advocate Christ Medical Center, 105 Covington Ct Oak Brook, IL 60523, USA; b Rosalind Franklin University of Medicine and Science, North Chicago, IL, USA; c Department of Cardiology, Advocate Christ Medical Center, 105 Covington Ct Oak Brook, IL 60523, USA

**Keywords:** *Blastomyces dermatitidis*, Immunosuppression, Orthotopic heart transplant, Opportunistic infection

## Abstract

Post-transplant immunosuppression is necessary to prevent organ rejection. Immunosuppression itself can introduce complications arising from opportunistic infections. We present a case of disseminated blastomycosis manifested only as a skin lesion in an asymptomatic patient post-orthotopic heart transplantation. A 64-year-old female who had recently undergone orthotopic heart transplant for end-stage ischemic cardiomyopathy presented for a scheduled routine cardiac biopsy. The patient had no current complaints other than a crusted plaque noticed at her nasal tip. It initially manifested 6 months after surgery as a pimple that she repeatedly tried to manipulate resulting in redness and crust formation. Her immunosuppressive and prophylactic medications included: mycophenolate, tacrolimus, prednisone, bactrim, acyclovir, valganciclovir, pyrimethamine/sulfadiazine, and fluconazole. On physical examination, she was flushed, with a large and exquisitely tender crusted necrotic lesion involving almost the entire half of the nose anteriorly, the left forehead and right side of the neck. She had decreased air entry over the right lung field as well. A computed tomography (CT) image of the chest was ordered to investigate this concerning physical exam finding in the post-transplant state of this patient on immunosuppressive therapy. Chest CT revealed bilateral nodular pulmonary infiltrates with confluence in the posterior right upper lobe. Blood cultures for aerobic and anerobic organisms were negative. Both excisional biopsy of the nasal cutaneous ulcer and bronchial biopsy demonstrated numerous fungal yeast forms morphologically consistent with *Blastomyces*. Cultures of both specimens grew *Blastomyces dermatitidis*, with methicillin-resistant *Staphylococcus aureus* (MRSA) superinfection of the nose. She received 14 days of intravenous (IV) amphotericin B for disseminated blastomycosis and 7 days of IV vancomycin for MRSA. Her symptoms and cutaneous lesions improved and she received maintenance itraconazole treatment for 1 year. This case illustrates a delicate balance that must be struck between suppressing the immune response to prevent graft rejection and avoiding over-immunosuppression that can lead to susceptibility to infection. Thus, in any post-transplant patient, a vigorous history and physical must be performed given that infections may present without symptoms and cause grave consequences.

## Introduction

Since 1954 with the advent of solid organ transplant, there has been a way to definitively treat end-organ damage. However, the surgery is just the first step. What follows is a long-term commitment on the part of both patient and physician to manage the new organ. One of the major challenges is to prevent the host body from rejecting the donor organ and in order to do so, the patient must strictly adhere to a group of medications that deliberately suppress the host’s immune system. Unfortunately, with this immunosuppressive therapy, the host is subject to constant bombardment from pathogens both common and opportunistic. Therefore, both physician and patient must remain vigilant in order to catch infections early before damage occurs.

Fungal organisms specifically cause a big threat to our patient population. Infections tend to be more severe than bacterial or viral and can sometimes be fatal. Recipients of solid organ and bone marrow transplants are predisposed to invasive fungal infections with Candida species, *Aspergillus*, and *Cryptococcus neoformans* [1]. Infections caused by *Blastomyces dermatitidis* are quite rare in transplant recipients [1]. What follows is a case of disseminated blastomycosis manifested only as a skin lesion in an asymptomatic patient post-orthotopic heart transplantation.

It is important for the clinician to be aware of the potentially serious consequences of opportunistic infections in patients that are over-immunosuppressed status post-transplant surgery despite being completely asymptomatic. We describe a rare case of a patient who developed systemic blastomycosis infection after an orthotopic heart transplantation.

## Case Report

**Figure 1:**
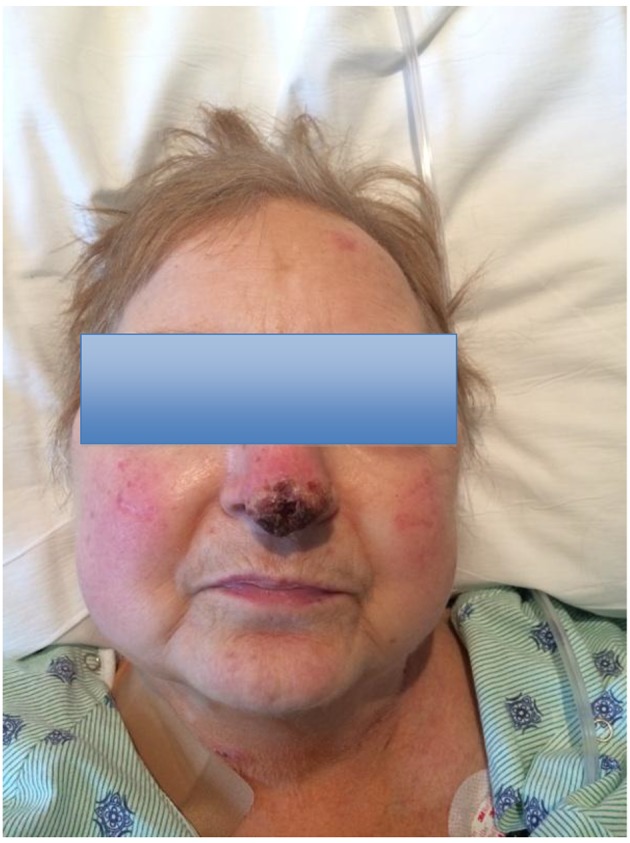
Photograph of the patient’s face showing the nose lesion on presentation to the hospital.

A 64-year-old female who had recently undergone orthotopic heart transplant for end-stage ischemic cardiomyopathy presented for a scheduled routine cardiac biopsy 6 months after her transplant. She had a long-standing history of coronary artery disease, bioprosthetic mitral valve replacement and cardiac arrest. In the postoperative cardiac recovery room, following the cardiac biopsy, the patient complained of a painful lesion on her nose. A crusted plaque was observed at the nasal tip (Fig. 1). She then reported squeezing the pimple 2 weeks prior to the myocardial biopsy and the following day, it became more swollen and involved. From here, the patient became more aggressive in squeezing this lesion on her nose which resulted in a dramatic increase in the amount of redness, swelling, and crusting at the tip of her nose. She tried multiple creams and ointments that provided no relief, then it was time for her myocardial biopsy, so she presented to the hospital.

Her immunosuppressive and prophylactic medications after the orthotopic heart transplant included: mycophenolate, tacrolimus, prednisone, bactrim, acyclovir, valganciclovir, pyrimethamine/sulfadiazine, and fluconazole. On physical exam, she was flushed, with a large and exquisitely tender crusted necrotic lesion involving almost the entire half of the nose anteriorly, the left forehead and right side of the neck. She had decreased air entry over the right lung field despite denying any respiratory symptoms.

**Figure 2:**
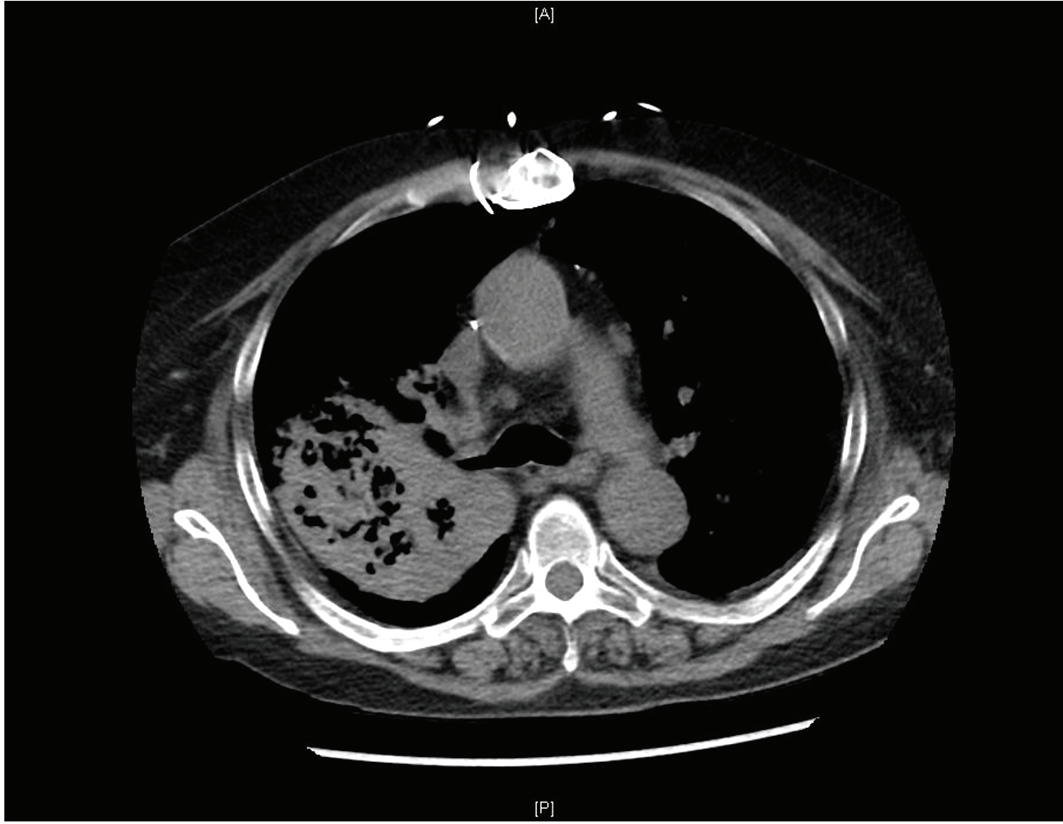
Initial chest CT of patient with bilateral nodular pulmonary infiltrates with predominance in the posterior right upper lobe.

Chest computed tomography (CT) displayed extensive bilateral nodular pulmonary infiltrates with confluence in the posterior right upper lobe (Fig. 2). Blood cultures for aerobic and anerobic organisms were negative. Both excisional biopsy of the nasal cutaneous ulcer and bronchial biopsy demonstrated numerous fungal yeast forms morphologically consistent with *Blastomyces*. Cultures of both specimens grew *Blastomyces dermatitidis*, with MRSA superinfection of the nose. She received 14 days of IV amphotericin B for disseminated blastomycosis and 7 days of IV vancomycin for MRSA. Her symptoms and cutaneous lesions improved and she received maintenance itraconazole treatment for 1 year.

## Discussion

Blastomycosis is a systemic disease caused by the dimorphic fungus *B. dermatitidis* that is seen mostly in the Ohio-Mississippi and St. Lawrence River areas of North America [2]. *B. dermatitidis* is usually benign in immunocompetent hosts presenting with mild respiratory symptoms. However, in those with weakened or compromised immune systems, *B. dermatitidis* can behave opportunistically creating a severe infection and increasing mortality rates as compared to infected immunocompetent hosts. The infection primarily presents with pulmonary symptoms such as cough, fever, sputum production, chest pain, hemoptysis, night sweats, weight loss and shortness of breath [3]. Chest radiographs will primarily show alveolar infiltrates or a mass lesion.

The second most commonly affected organ that is infected is the skin. The characteristic verrucous lesion has irregular borders and may range in color from gray to purple. Furthermore, these lesions can appear anywhere on the skin. Microscopic examination will show broad based budding yeasts characteristic of *B. dermatitidis* [4]. Other sites of infection for blastomycosis include the skeletal system, the genitourinary system, and the central nervous system. However manifestations in these systems are rare and occur in 4%, 2%, and 1% respectively in patients infected with blastomycosis.

After a literature review of post-transplant blastomycosis infections, our research has found that blastomycosis infection is quite rare in this patient population. A 2007 retrospective review by Gauthier et al found that between 1986 and 2004 there were only 11 documented cases of post-transplant blastomycosis out of 8,104 transplant surgeries, an incidence of 0.14%. Another retrospective review published in 2011 by Grim et al found that between 1996 and 2008 only eight cases of post-transplant blastomycosis were identified out of a possible 5,989 transplant operations, a similar incidence of 0.13%. However, the incidence of blastomycosis infection is not the only aspect that makes this case so rare. Of the 19 cases found in the two reviews mentioned previously, none of the patients who became infected with *B. dermatitidis* had undergone a heart transplant. In the review performed by Grim et al, all eight patients had received renal transplants and in the review by Gauthier et al, seven patients underwent renal transplants, three patients had liver transplants, and one case was a lung transplant. Furthermore, our patient presented with signs of infection 6 months after surgery, Gauthier et al found the median time to first infection to be 26 months. Our patient’s initial complaint of skin lesion was also unusual. Blastomycosis infection most commonly presents with infections involving the lungs as documented by the review performed by Gauthier et al which revealed that in 82% (9/11) of the cases, pneumonia was the presenting diagnosis. Cutaneous lesions, however, is often not an initial symptom of disease but rather that of disease dissemination. Gauthier et al found disease dissemination in 36% of cases (4/11), but of these four cases, three of them had various cutaneous lesions. Grim et al found that 50% (4/8) of their blastomycosis cases had skin involvement. So while cutaneous lesions are often seen with advancing infection, it is rarely the chief complaint.

The primary means of treatment for blastomycosis infection in immunocompromised patients is amphotericin B therapy for a maximum of 2 weeks at which point therapy is transitioned to itraconazole. Patients for whom immunosuppression is irreversible, itraconazole therapy should be a chronic treatment because relapse is a common problem in this patient population.

Treatment for our patient’s disseminated blastomycosis infection required delicacy as to not damage the transplanted heart but at the same time to adequately treat the infection. Deciding whether to favor immunosuppression or increased patient humoral immunity must be taken on a case-by-case basis. In our patient, we ultimately decided to tip the balance towards less immunosuppression. We accomplished this by decreasing the tacrolimus and mycophenolate dosages while maintaining the prednisone dose. Voriconazole was stopped and amphotericin B was started in its place. Additionally, azithromycin and imipenem were discontinued.

This case illustrates the delicate balance that must be struck between suppressing the immune response to prevent graft rejection and avoiding over-immunosuppression that can lead to susceptibility to infection. Thus, in any post-transplant patient, clinicians should be diligent in their assessment for infections because the patient may not present with any symptoms. Quick diagnosis and aggressive treatments are keys to fighting infection, but immunosuppressed patients present many more challenges to diagnosis and therefore treatment. Immunosuppressed patients will often have dampened initial responses to infection, with unreliable physical signs and laboratory findings, causing the diagnosis to be quite challenging. Consequently, immunocompromised patients usually present with atypical symptoms and with more disseminated infections at presentation. To further complicate the situation, a weakened immune system increases the number of possible sources due to the inclusion of opportunistic infections. All of these factors together create a challenge to the physician specifically in diagnosis and treatment of infection in immunocompromised patients [4].

### Conclusion

Transplant patients must be treated with anti-rejection medications in order to ensure the survival of their new organ. As such, it is the job of both patient and provider to examine any signs or symptoms that could be a manifestation of infection no matter how small or how benign they seem to be.
